# Inter- and intra-individual variability in alpha peak frequency

**DOI:** 10.1016/j.neuroimage.2014.01.049

**Published:** 2014-05-15

**Authors:** Saskia Haegens, Helena Cousijn, George Wallis, Paul J. Harrison, Anna C. Nobre

**Affiliations:** a Department of Psychiatry, Columbia University College of Physicians and Surgeons, New York, USA; b Cognitive Neuroscience and Schizophrenia Program, Nathan Kline Institute, Orangeburg, USA; c Department of Psychiatry, Warneford Hospital, University of Oxford, Oxford, UK; d Oxford Centre for Human Brain Activity, University of Oxford, Oxford, UK; e Department of Experimental Psychology, University of Oxford, Oxford, UK

**Keywords:** Alpha, Beta, Oscillations, MEG

## Abstract

Converging electrophysiological evidence suggests that the alpha rhythm plays an important and active role in cognitive processing. Here, we systematically studied variability in posterior alpha peak frequency both between and within subjects. We recorded brain activity using MEG in 51 healthy human subjects under three experimental conditions — rest, passive visual stimulation and an N-back working memory paradigm, using source reconstruction methods to separate alpha activity from parietal and occipital sources. We asked how alpha peak frequency differed within subjects across cognitive conditions and regions of interest, and looked at the distribution of alpha peak frequency between subjects. In both regions we observed an increase of alpha peak frequency from resting state and passive visual stimulation conditions to the N-back paradigm, with a significantly higher alpha peak frequency in the 2-back compared to the 0-back condition. There was a trend for a greater increase in alpha peak frequency during the N-back task in the occipital vs. parietal cortex. The average alpha peak frequency across all subjects, conditions, and regions of interest was 10.3 Hz with a within-subject SD of 0.9 Hz and a between-subject SD of 2.8 Hz. We also measured beta peak frequencies, and except in the parietal cortex during rest, found no indication of a strictly harmonic relationship with alpha peak frequencies. We conclude that alpha peak frequency in posterior regions increases with increasing cognitive demands, and that the alpha rhythm operates across a wider frequency range than the 8–12 Hz band many studies tend to include in their analysis. Thus, using a fixed and limited alpha frequency band might bias results against certain subjects and conditions.

## Highlights

• Alpha peak frequency increases with cognitive demand.
• No strict harmonic relationship between alpha and beta.
• Using a limited and fixed band for alpha biases against certain subjects/conditions.

## Introduction

The prominent posterior alpha rhythm was first recorded by Hans Berger (1929) and long considered to reflect cortical idling (Adrian and Matthews, 1934; Pfurtscheller et al., 1996). More recently, converging electrophysiological evidence suggests that the alpha rhythm actually plays an important and active role in cognitive processing (Cooper et al., 2003; Jensen and Mazaheri, 2010; Klimesch et al., 2007). In particular, alpha oscillations are proposed to reflect a mechanism of functional inhibition (Foxe and Snyder, 2011; Jensen et al., 2012; Mathewson et al., 2011), regulating the engagement and disengagement of sensory areas depending on task demands.

In support of this idea, several studies have shown that alpha oscillations reflect the focus of attention, both in the visual (Gould et al., 2011; Thut et al., 2006; Worden et al., 2000) and the somatosensory system (Anderson and Ding, 2011; Haegens et al., 2011a; Jones et al., 2010), with consequences for subsequent perceptual performance. Furthermore, alpha activity has been shown to increase with load during working memory (WM) maintenance, presumably in order to facilitate WM retention by preventing interfering inputs (Jensen et al., 2002; Sauseng et al., 2009; Tuladhar et al., 2007).

Alpha peak frequency is known to change with age, increasing up to adulthood and then decreasing with older age (Aurlien et al., 2004; Lindsley, 1939). Inter-subject variability in alpha frequency is to a large degree explained by genetic factors (e.g., Bodenmann et al., 2009), with twin studies showing heritability estimates of about 80% (Smit et al., 2006; Van Beijsterveldt and Van Baal, 2002). Inter-subject differences in alpha peak frequency have been linked to various cognitive measures, including WM performance (reviewed in Klimesch, 1999). Additionally, intra-subject variability in alpha peak frequency has been described, which may reflect different alpha networks kicking in dependent on task demands (Başar, 2012; Klimesch, 1999).

Thus, alpha frequency can be seen both as a ‘trait’ variable, with inter-subject variability potentially explaining differences in overall cognitive performance, as well as a ‘state’ variable, with intra-subject variability possibly reflecting fluctuations in moment-to-moment performance. Knowing the range within which the posterior alpha rhythm operates, both between and within subjects, will be crucial in order to interpret results from studies that try to explain performance differences in terms of alpha activity modulations.

However, most studies define the alpha rhythm as a fixed narrow band (most commonly 8–12 Hz), and average over spectral activity within that fixed band for all subjects. It has been argued that using the individual alpha frequency (IAF), determined per subject (defined in terms of either peak or ‘gravity’ frequency), gives a more accurate estimate of alpha modulated activity (Doppelmayr et al., 1998; Klimesch, 1999; although see Smit et al., 2005; Shackman et al., 2010). The reasoning is that because of substantial inter-individual variability in alpha frequency (a mean SD of 1 Hz is reported, cf. Klimesch, 1997), significant portions of alpha power will fall outside a fixed frequency window, and/or activity from neighboring frequencies (i.e., theta or beta) might erroneously be included in the fixed alpha window. Along these same lines, it was suggested that the alpha frequency range should be further subdivided into low- and high-alpha subranges, which may behave differently under certain task conditions (Klimesch et al., 1996, 1998). While adopted by a substantial part of the field, this approach is by no means common practice. Given the emergence of more sensitive analyses of especially alpha phase (e.g., cross-frequency coupling mechanisms, phasic modulation of stimulus processing), optimized individual peak frequency detection might become essential.

Here, we systematically studied how posterior alpha peak frequency varies both between and within subjects. We aimed to establish whether individual alpha peak variability indeed goes beyond the often-used 8–12 Hz fixed band. We recorded brain activity using MEG in 51 healthy human subjects under three experimental conditions — rest, passive visual stimulation and an N-back WM paradigm. Using MEG in combination with source reconstruction methods allowed us to separate alpha activity from parietal and occipital sources, which to the best of our knowledge has not been done before in this context. We asked how alpha peak frequency differed within subjects across cognitive conditions and regions of interest, and looked at the distribution of alpha peak frequency across this relatively large set of subjects. Furthermore, we explored the relation between individual alpha and beta peak frequencies, as a harmonic relationship between the two has been suggested (Carlqvist et al., 2005; Gaarder and Speck, 1967; Klimesch, 2012).

## Methods

### Participants

Fifty-one healthy right-handed volunteers (27 female, 24 male; mean age 24.2 years; range 19–34) with normal or corrected-to-normal vision participated in this experiment. Ethical approval was obtained from the NHS South Central Berkshire ethics committee (11/SC/0053). Each subject participated in three experimental blocks that were recorded successively: (1) resting state, (2) N-back, (3) visual gratings.

### Paradigm

Resting state: 6 min of resting state was recorded while subjects kept their eyes open and fixated on a fixation cross.

**Fig. 1:**
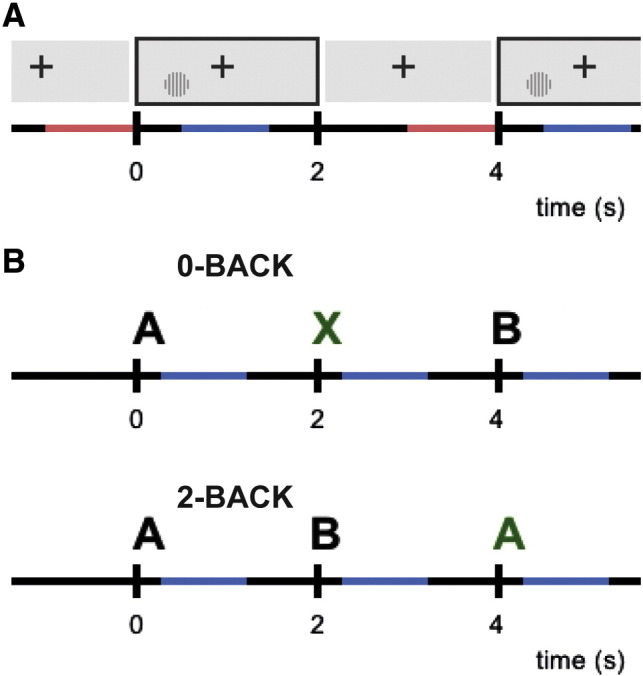
Experimental paradigm. (A) Visual condition: stimuli consisting of vertical, stationary gratings were presented in either the left or right lower visual field, while participants maintained central fixation. Stimuli were presented for 2 s followed by a 2-s baseline window. Analysis windows (1 s length) are indicated in blue for stimulation and in red for baseline. (B) N-back task: stimuli consisting of letters were presented for 200 ms at 2-s SOA. In the 0-back task (upper panel) the subject had to respond to the letter X. In the 2-back task the subject had to respond if the letter was the same as that of two stimuli back. (Targets are presented in green here for illustrative purposes only.) Analysis windows (1 s length) are indicated in blue on the time axis.

Visual gratings (Fig. 1A): stimuli consisting of vertical, stationary, maximum-contrast, 3-cycles-per-degree gratings were presented on a mean luminance background. Ninety stimuli were presented in either the left or right lower visual field. Participants were instructed to maintain fixation on a dot in the middle of the screen for the duration of the experiment. Stimuli were presented for 2 s followed by 2 s of fixation in blocks of five, with each block of five followed by 20-s fixation during which the subjects were allowed to blink.

N-back (Fig. 1B): the N-back paradigm consisted of a 0-back and a 2-back task, seven blocks each, presented in alternating fashion, followed by 15-s breaks. Each block consisted of presentation of 15 letters with 200-ms stimulus duration and 2-s SOA, i.e., 1.8-s WM retention/decision period. Each block contained 2–4 targets. In the 0-back task, subjects had to respond by button press to the letter X, while on the 2-back task subjects had to respond when the stimulus was the same as the one two stimuli back.

### Data acquisition

Whole-head MEG recordings were acquired at a sampling frequency of 1000 Hz, using an Elekta NeuroMag MEG System. Data from the 204 gradiometers were analyzed. A magnetic digitizer (Polhemus FastTrach 3D) was used to measure the relative positions of four head-position indicator coils and three anatomical landmarks (nasion, left and right auricular points). These coordinates were used for co-registration of the sensor montage to the participant's anatomical magnetic resonance image (MRI), which was acquired using a 3 T Siemens system.

### Data analysis

The data were analyzed using custom-build Matlab code, the FieldTrip toolbox for EEG/MEG-analysis (Oostenveld et al., 2011; http://www.ru.nl/neuroimaging/fieldtrip/) and SPM8 (http://www.fil.ion.ucl.ac.uk/spm). The data were down-sampled offline to a sampling frequency of 500 Hz, after applying a 0.5 Hz high-pass filter and a 200 Hz low-pass filter. Bad channels and trials were rejected upon visual inspection. We used independent component analysis (Jung et al., 2000) to identify eye artifacts, which were then projected out of the data.

First, we studied the main effects in each condition by computing sensor level power spectra and whole brain source reconstructions, focusing on alpha activity within the band of 7–14 Hz. Second, we determined each subject's individual alpha peak frequency for each condition and region of interest (ROI), using a time-domain beamformer approach.

#### Spectral analysis

Power spectra were calculated on 1-s segments of data for each condition: (1) for the resting state, the continuous recording was segmented into 1-s epochs; (2) for the visual gratings a 1-s window prestimulus (t = − 1–0 s) and a 1-s window during stimulus (t = 0.5–1.5 s; i.e., 500 ms after stimulus onset to exclude the initial evoked response); and (3) for the N-back task, a 1-s window during the retention interval, after stimulus offset (t = 0.2–1.2 s) was selected. The analyses included on average 340 trials (range, 298–350) per subject for rest; 86 (72–90) for baseline and for stimulus; 101 (83–105) for 0-back and 102 (82–105) for 2-back. These segments were zero-padded to 10 s and multiplied with a Hanning taper, and power of 4–30 Hz was computed using a fast Fourier transform (FFT) approach. To inspect the time course of the frequency effects, we also computed time–frequency representations (TFRs) of the power spectra for the full trials per experimental condition. To this end we used an adaptive sliding time window of four cycles length (Δt = 4 / f) for each frequency represented and applied a Hanning taper before estimating the power using an FFT approach.

#### Source analysis

To localize the sources of the alpha band activity for each condition, we applied a frequency-domain beamformer (Gross et al., 2001; Schoffelen et al., 2008). This adaptive spatial filtering technique uses the Fourier spectra, which were obtained by applying a multitaper FFT approach to the 1-s data segments, centered at 10.5 Hz with six orthogonal Slepian tapers resulting in ± 3.5 Hz smoothing (Percival and Walden, 1993), i.e., a band of 7–14 Hz. We constructed a realistically shaped single-shell description of the brain for each subject, using the individual anatomical MRI. The brain volume of each individual subject was divided into a grid with a 1 cm resolution and normalized toward the template Montreal Neurological Institute (MNI) brain (International Consortium for Brain Mapping, Montreal Neurological Institute, Canada) using SPM8. Lead fields were calculated for all grid points (Nolte, 2003). With the lead fields and the Fourier spectra (per paradigm, per subject) a spatial filter was constructed for each grid point (note that within the visual paradigm a common spatial filter was used for baseline and stimulus windows, and within the N-back paradigm a common spatial filter was used for 0-back and 2-back). Using this filter, the spatial distribution of power was estimated for each trial separately, and then averaged per condition and contrasted in order to reduce the center bias (visual: stimulus vs. baseline; N-back: 2-back vs. 0-back; resting state: the lead fields were normalized to obtain the bias reduction, since there was no condition to contrast with) to reveal the sources of the alpha band activations.

Note: for one subject a standard brain template was used, as no MRI was available for this subject.

#### Statistical analysis

Statistical analysis was performed both on sensor and source levels, using the same procedure. For each subject, power spectra were normalized by dividing with average power in the spectrum per condition (for each sensor/grid point separately). This procedure reduces inter-subject variability in the power estimates. We then tested for each experimental condition whether there was a significant alpha band modulation, using the following contrasts: 1) visual paradigm: stimulus vs. baseline; 2) N-back paradigm: 2-back vs. 0-back; 3) resting state: rest vs. pseudo-estimate (i.e., since there was no condition to contrast with, we tested alpha against the average power in the spectrum). To establish whether these contrasts were significantly different from 0, a cluster-based nonparametric randomization test was applied (Maris and Oostenveld, 2007). By clustering neighboring sensors (or grid points in the source analysis) that show the same effect, this test deals with the multiple-comparisons problem and at the same time takes into account the dependency of the data. The normalized data were averaged over the alpha frequency range (7–14 Hz) and for each sensor a dependent-samples t value was computed. All samples were selected for which this t value exceeded an a priori threshold (uncorrected p = 0.05), and these were subsequently clustered on the basis of spatial adjacency. The sum of the t values within a cluster was used as cluster-level statistic. The cluster with the maximum sum was subsequently used as test statistic. By randomizing the data across the two conditions and recalculating the test statistic 2000 times, we obtained a reference distribution of maximum cluster t values to evaluate the statistic of the actual data.

#### Source-level peak detection

Next, in order to establish each subject's individual alpha peak frequency across conditions and ROIs, we applied a linearly constrained minimum variance (LCMV) beamformer technique to extract source-reconstructed frequency spectra from the parietal and the occipital lobe. ROIs were defined based on an anatomical atlas (Talairach and Tournoux, 1988). Spheres with radius of 2.5 cm were centered at left and right Brodmann area (BA) 7 for the parietal ROI, and similar spheres were placed so to include both BA 17 and 18 for the occipital ROI.

The LCMV beamformer is a time-domain beamformer, and the spatial filters are computed using the covariance-matrix, which was calculated between all sensor pairs on the low-pass filtered (30 Hz, FIR) 1-s data segments per condition (the data were epoched as described above, Spectral analysis section). Lead fields were computed as described above (Source analysis section). Spatial filters were computed for each subject, similarly as described above for the frequency-domain beamformer (i.e., one filter for resting state, a common filter for baseline and visual stimulus, and a common filter for 0-back and 2-back), and applied to reconstruct virtual channel time-courses for both ROIs (occipital and parietal), for each condition: rest, baseline, visual stimulus, 0-back and 2-back.

We then computed frequency spectra (4–30 Hz) on this virtual-channel data using the same FFT approach as we used on sensor level. To determine the subject's peak frequency, we detected the highest local maximum within the 7–14 Hz band with a .1 Hz resolution, per ROI and condition. If no peak was present, it was set to Nan. Additionally, we used an adaptive algorithm fitting a Gaussian curve to the power spectra and used these for estimating the individual alpha peak frequency (cf. Van Albada and Robinson, 2013), in order to confirm our original peak detection. This approach may give more accurate peak estimates, e.g., in case the spectrum contains two local maxima close to each other in the alpha range, or in case of noisy spectra with spurious peaks, as it effectively smoothes the spectra. Furthermore, it is a more conservative approach as subjects without substantial modulation in the alpha range are automatically omitted (i.e., Gaussian fit fails), allowing us to verify that our initial analysis was not biased by inclusion of potentially spurious peaks.

In order to detect the beta peak frequency, we first removed the 1/f component of the spectrum, as this obscures the smaller peaks in the beta range (14–30 Hz) by strongly biasing lower frequencies. In order to compensate for the 1/f effect, linear regression (least-squares fit) was used to fit a linear model to the log-transformed spectrum in the beta range. The fitted linear trend was then subtracted from the spectrum, allowing for a more reliable beta peak frequency estimate (cf. Nikulin and Brismar, 2006).

## Results

MEG data were acquired in 51 subjects, for (1) resting state, (2) passive visual stimulation, and (3) an N-back paradigm.

### Main effect: widespread posterior alpha across conditions

**Fig. 2:**
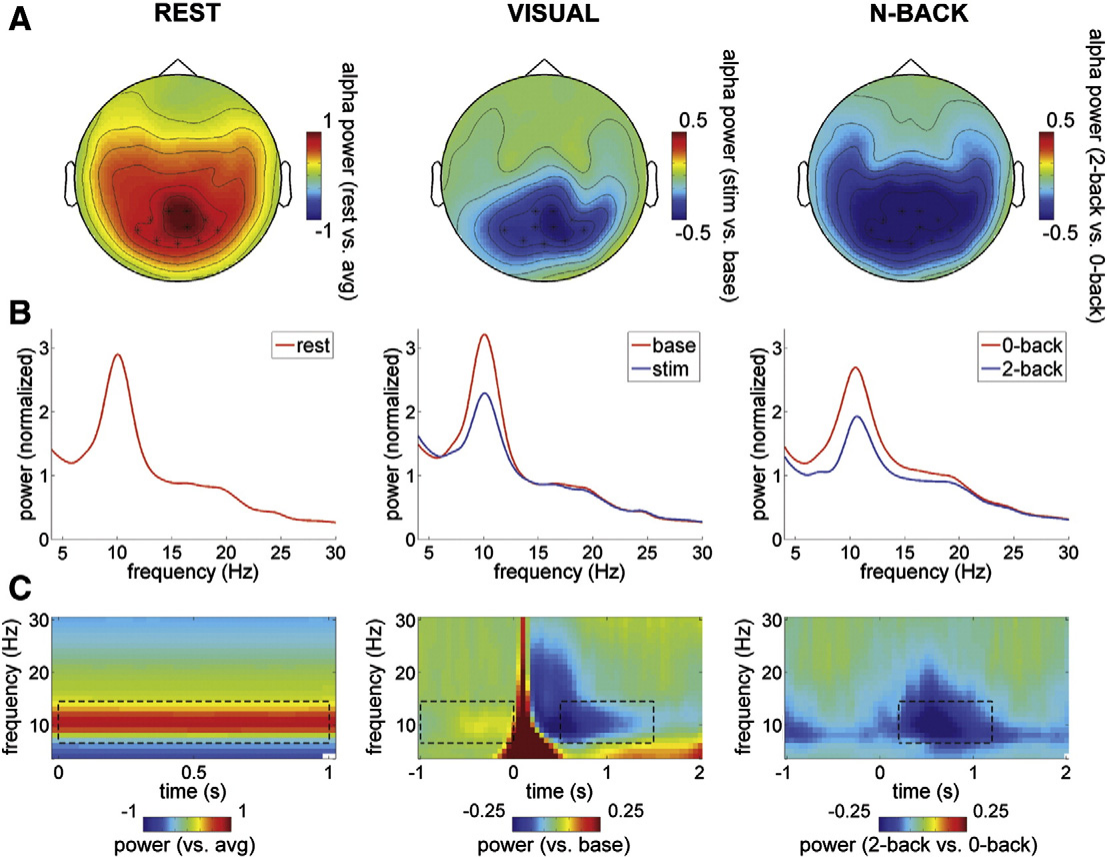
Alpha activity per condition on sensor level. (A) Topographic plots showing alpha power (7–14 Hz) modulation per condition, computed on 1-s epochs (rest: alpha power vs. average power in the spectrum [arbitrary 1-s segments]; visual: stimulus [t = 0.5–1.5 s] vs. baseline [t = − 1–0 s]; N-back: 2-back vs. 0-back during retention interval [t = 0.2–1.2 s]). Time–frequency windows used are indicated in C with dashed lines. (B) Power spectra (averaged over sensors marked with asterisks in A) for each of the conditions, showing clear peaks in the alpha band. (C) TFRs showing alpha power modulation per condition (averaged over sensors marked in A). Rest: alpha power increase as compared to average spectral power (arbitrary 1-s epochs); visual: decrease of alpha power during stimulus (t = 0–2 s) as compared to baseline (t = − 1–0 s); N-back: decrease of alpha power for 2-back compared to 0-back (stimulus: t = 0–0.2 s, retention: t = 0.2–2 s). All plots showing grand-averages over 51 subjects.

First, we analyzed the main effect of alpha power modulation (7–14 Hz) for each of the conditions on sensor-level data. We calculated the power spectra (4–30 Hz) on 1-s epochs for the following conditions: (1) resting state, (2) baseline and stimulus window for the visual gratings paradigm, and (3) 0-back and 2-back for the N-back paradigm (Figs. 2A,B). Per subject, the spectra were normalized per condition with the average power across frequencies. The sensor-level spectral analysis revealed a peak in the alpha band for each condition, with maximum activity located in posterior sensors. The sensor-level alpha power modulation was significant for all conditions (cluster-based randomization test, p < 0.01 for visual, and p < 0.001 for rest and N-back).

A time–frequency analysis showed a sustained increase of alpha power during rest as compared to average spectral power, a decrease of alpha during visual stimulation as compared to pre-stimulus baseline activity, and a decrease of alpha during the 2-back as compared to the 0-back task (Fig. 2C). The alpha modulation appeared strongest right after the offset of the initial evoked response to the visual stimulus, and after offset of the stimulus for the N-back paradigm.

**Fig. 3:**
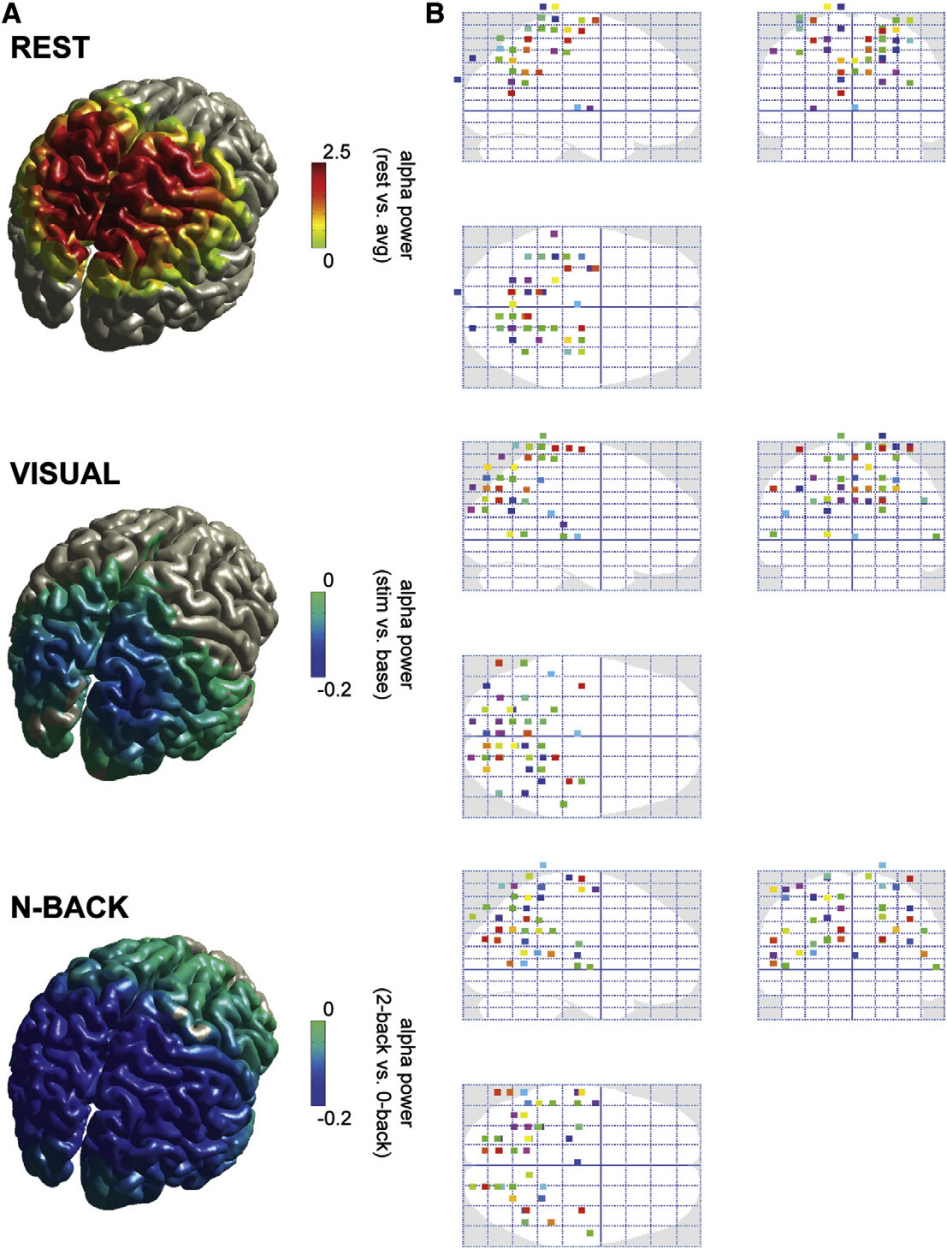
Alpha activity per condition on source level. (A) Alpha power source reconstructions obtained using beamformer technique, shown on a standardized brain volume, per condition (contrasts same as in Fig. 2A). Power values were masked to only show grid points with significant alpha power modulation (cluster-based randomization test, p < 0.05), showing grand-average over 51 subjects. (B) Glass brain projections of peak activity per subject (N = 51), per condition. For each subject, the peak location of alpha activity was localized using the beamformer source reconstruction, and projected to Talairach space. (Note that points appearing slightly outside the brain here are due to inaccuracies introduced by normalizing the individual head model to that of standard brain in MNI space, and subsequently converting these coordinates to Talairach space, in addition to using a grid with 1-cm resolution.)

Using a beamformer approach, the sources of alpha activity (7–14 Hz) could be localized for each of the conditions (Fig. 3A). Based on the source reconstructions averaged over 51 subjects, the resting state alpha seemed somewhat more anterior than the visually induced alpha which was more posterior localized, while N-back modulated alpha was fairly widespread. All conditions had a posterior-parietal dominance (cluster-based randomization test, p < 0.001 for all conditions). Comparing the locations of peak activity for each subject across conditions, confirms the widespread origin of the alpha activity, including occipital and parietal sources (Fig. 3B).

### Individual alpha peak frequency

**Fig. 4:**
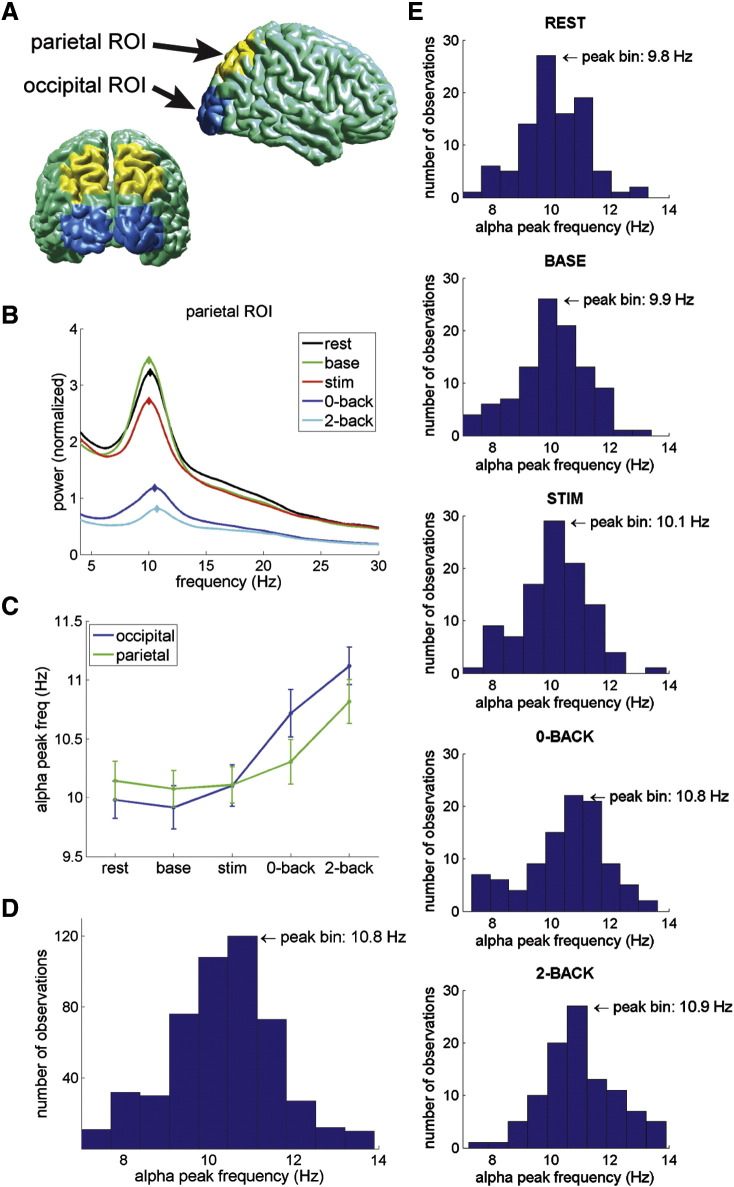
Individual alpha peak frequency. (A) Projection of the parietal and occipital bilateral ROIs onto standard brain surface. (B) Power spectra per condition for the parietal ROI, averaged over 51 subjects (normalized per subject with average power in all conditions). (C) Alpha peak frequency per condition per ROI, showing average over all subjects that had a discernible alpha peak for that condition ∗ ROI. Error bars indicate the SEM. Significant increase of alpha peak frequency from rest/baseline/stimulus to 0-back, and from rest/baseline/stimulus/0-back to 2-back. (D) Histogram showing distribution of individual alpha peak frequencies, including all measurements. Total number of observations was 510 (i.e., 2 ROIs, 5 conditions, 51 subjects). Note: number of observations of undetectable peak frequency was 11, not shown in histogram. Bin with highest number of observations was centered at 10.8 Hz. (E) Histograms per condition for both ROIs combined, showing increase of alpha peak frequency and shift of distribution from left (lower alpha peak frequency) to right (higher alpha peak frequency).

**Fig. 5:**
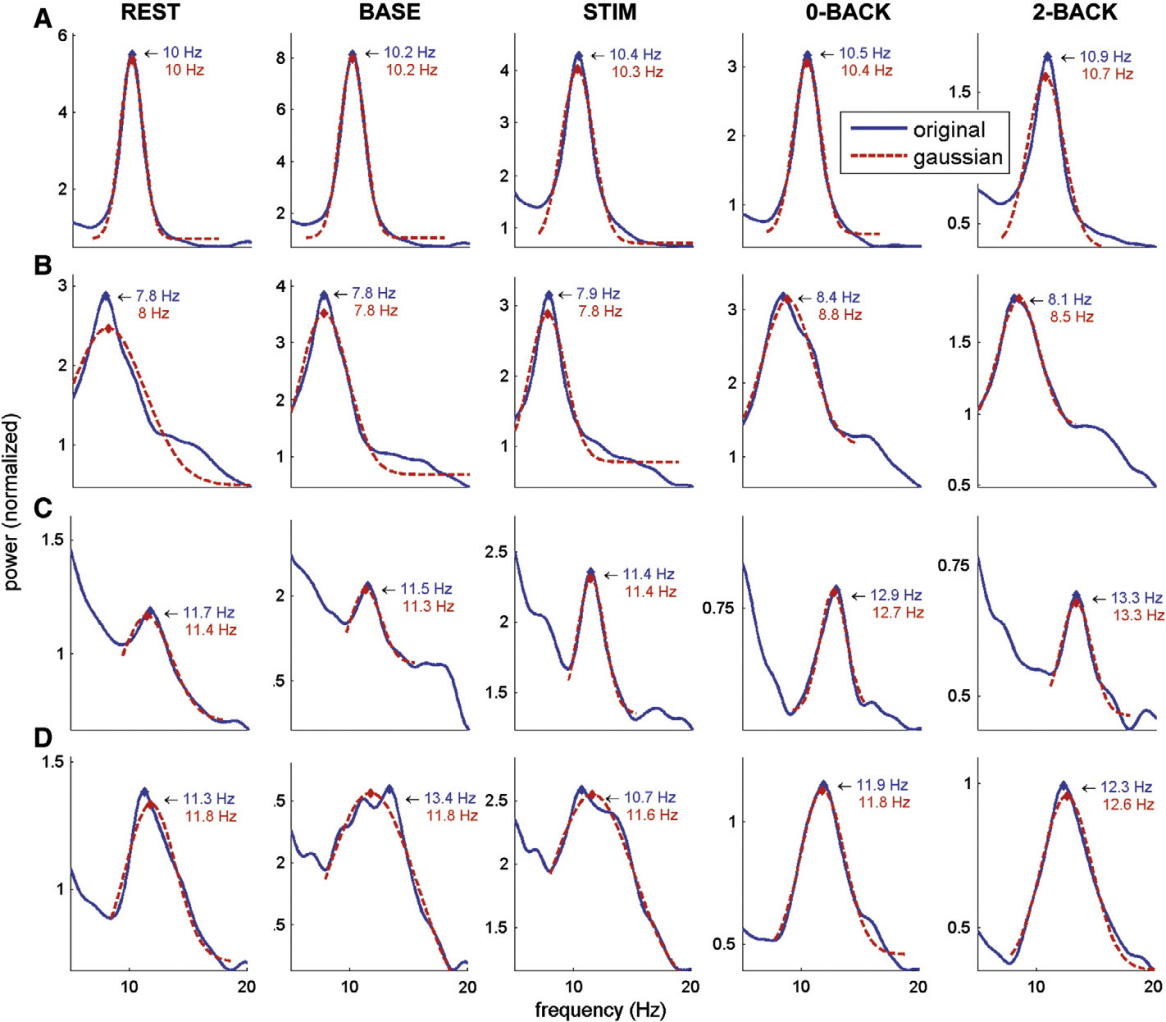
Single subject examples of alpha peak frequency detection. Power spectra showing alpha peak frequency detection on both the original ‘raw’ spectra (blue) and the Gaussian fit (red), per condition, for four representative subjects. Showing example plots from (A) subject 5, parietal ROI; (B) subject 8, parietal ROI; (C) subject 11, occipital ROI; (D) subject 18, occipital ROI.

Having established alpha band activity in each of the conditions, we proceeded to determine each individual's alpha peak frequency for two ROIs, occipital and parietal cortex (Fig. 4A), for each of the conditions (Fig. 4B, showing grand-average spectra per condition for the parietal ROI). The individual alpha peak frequency detection was performed within the predefined alpha band of 7–14 Hz, and defined as the biggest local maximum within that range (refer to Fig. 5 for examples from four representative subjects). The alpha peak frequencies increased for the N-back compared to the other conditions, and were fairly similar across ROIs with a deviation during the N-back task (Table 1, Fig. 4C).

#### Within-subject variability

We computed a two-way repeated-measures ANOVA with factors condition (rest, baseline, stimulus, 0-back, 2-back) and ROI (occipital, parietal) to assess variability of the alpha peak frequency within subjects. There was a significant effect of condition (F(4,180) = 26.485, p < 0.001) and no effect of ROI (F(1,45) = 0.939, p = 0.338) on alpha peak frequency. Although there appeared to be a trend, there was no significant interaction between condition and ROI (F(2.731,122.899) = 1.880, p = 0.142; reporting Greenhouse–Geisser correction as Mauchly's test showed that assumption of sphericity was violated, p < 0.05). Note that 46 subjects were included in the ANOVA analysis; the five excluded subjects had at least one missing alpha value. We further assessed the significant effect of condition by performing post-hoc pairwise comparisons, which showed a significant difference for rest, baseline and stimulus vs. 0-back (Bonferroni corrected p < 0.01) and 2-back (Bonferroni corrected p < 0.001), and between 0-back and 2-back (Bonferroni corrected p < 0.05).

These results were confirmed by estimating the individual alpha peak frequency using a Gaussian fit (refer to Fig. 5 for example fits for four representative subjects). In cases with a strong, unambiguous alpha peak, the Gaussian fit leads to virtually similar estimates as our original approach, whereas in other cases, for instance where alpha consists of a broader or double peak (see e.g. baseline and stimulus for subject 18, Fig. 5D), a more reliable estimate incorporating the full alpha range is computed. The Gaussian-fit approach is more conservative and excludes more cases, as noisy ambiguous peaks that cannot be fitted are omitted. Consequently, only 38 subjects were included (i.e., Gaussian curve fitting failed in the remaining instances). Using this conservative approach and applying the same two-way repeated-measures ANOVA, we again found a significant effect of condition (F(2.557,94.607) = 39.132, p < 0.001, Greenhouse–Geisser corrected) and no effect of ROI (F(1,37) = 0.242, p = 0.626). Now the condition ∗ ROI interaction was significant (F(2.090,77.324) = 3.706, p < 0.05, Greenhouse–Geisser corrected). Post-hoc tests confirmed a significant difference for rest, baseline and stimulus vs. 0-back and 2-back (Bonferroni corrected p < 0.001), and a trend for 0-back vs. 2-back (Bonferroni corrected p = 0.068). Additionally, the correlation between our original estimates and those based on Gaussian fit was significant (R^2^ = 0.739, p < 0.001), while a paired-sample t-test showed no significant difference between the two estimates (t(479) = 0.165, p = 0.869).

To further establish the reliability of our original alpha peak detection, we split the dataset in two parts (median split, taking per subject and condition the first vs. second half of trials) and repeated the alpha peak frequency detection on each set of trials. We performed a repeated-measures ANOVA, similar as before but now with the added factor time (first, second half). The effect of time was not significant (F(1,45) = 2.167, p = 0.148), while our previous results were confirmed: significant effect of condition (F(3.131,140.907) = 35.033, p < 0.001, Greenhouse–Geisser corrected), no effect of ROI (F(1,45) = 0.056, p = 0.814). None of the interaction effects was significant (p > 0.05).

Thus, alpha peak frequency increased significantly from the resting state and passive visual stimulation conditions to the N-back paradigm, with a further increase of alpha peak frequency from the 0-back to 2-back condition. Note that the latter effect was significant in our initial analysis, and was confirmed by a statistical trend in the more conservative Gaussian fit approach. No conclusive significant difference was detected between occipital and parietal alpha peak frequency, although the N-back increase seemed slightly stronger in occipital than parietal ROI.

#### Between-subject variability

Next, we investigated the distribution of individual alpha peak frequencies between subjects (Table 1, Fig. 4D). Taking all conditions and ROIs into account, detected alpha peak frequencies spread the full 7–14 Hz search range. The bin with the highest number of observations was centered at 10.8 Hz. The mean alpha peak frequency across subjects was 10.3 Hz with a between-subject SD of 2.8 Hz (compare with a within-subject SD of 0.9 Hz), and the median was 10.4 Hz. Inspecting the histograms per condition shows how the peak bin shifts to the right (higher alpha peak frequency) and the entire distribution becomes more rightward skewed (Fig. 4E).

### Beta peak frequency in relation to alpha

**Fig. 6:**
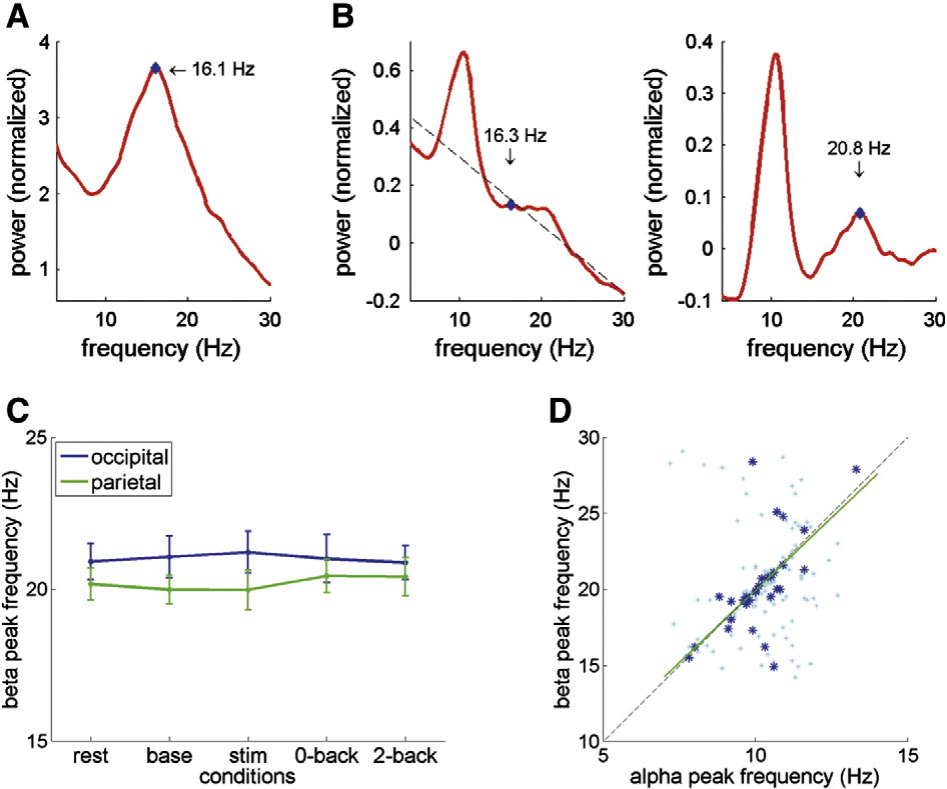
Individual beta peak frequency. (A) Power spectrum of one example subject who did not have an alpha peak, but did show a clear beta peak (subject 50, parietal ROI during rest). (B) Log-transformed power spectra showing procedure for beta peak detection using linear fit (dashed line) to remove 1/f component from spectrum in order to reliably estimate the beta peak (showing example for subject 39, parietal ROI during rest). Note that in the uncorrected spectrum a local maximum was found at 16.3 Hz (left panel), whereas after 1/f subtraction the beta peak is detected at 20.6 Hz (right panel). (C) Beta peak frequency per condition per ROI, showing average over all subjects that had a discernible beta peak for that condition ∗ ROI. Error bars indicate the SEM. No significant difference in beta peak frequency across conditions or ROIs. (D) Scatterplot (dark blue data points) showing positive correlation between alpha and beta peak frequencies for parietal ROI during rest (linear regression model in green; dashed line indicates beta = alpha ∗ 2, i.e., purely harmonic relationship). Additionally showing scatterplot for all other conditions (light blue data points).

While almost all subjects had clear alpha peaks in their spectra (although not necessarily for each condition and ROI), there were two subjects that even upon visual inspection of their spectra lacked a systematic alpha peak (Fig. 6A). However, both these subjects did have a distinct beta band peak. Additionally, the majority of subjects (approximately 2/3) that did have a clear alpha peak also showed a, usually much smaller, beta peak (Fig. 6B). We explored the potential harmonic relationship between alpha and beta by detecting for each subject (if present) the beta peak frequency in the 14–30 Hz range, for all conditions and ROIs. First, we tested whether there was any effect of condition or ROI on beta peak frequency (Fig. 6C). Since there were many more ‘missing values’ here (bar one subject, all had some condition ∗ ROI where no beta peak could be detected), rather than using the repeated-measures ANOVA as we did for the alpha peak frequency, we now applied a one-way ANOVA treating each condition ∗ ROI as a separate ‘group’. This showed no significant difference in beta peak frequency across conditions or ROIs (F(9,294) = 0.58, p = 0.82).

Having found no systematic variability in beta peak frequency across conditions and ROIs, we then asked how beta peak frequency related to alpha peak frequency. There was a significant positive correlation between alpha and beta peak frequency during rest in the parietal ROI (R^2^ = 0.402, Bonferroni corrected p < 0.001, N = 32; Fig. 6D). We found no significant correlation between alpha and beta peak frequencies in any of the other conditions, nor for the occipital ROI (Bonferroni corrected p > 0.1 for all; not shown). We then further explored the relation between alpha and beta peaks during rest in the parietal ROI. Comparing alpha and beta peaks, there was a difference of factor 1.995 (SD, 0.235; range, 1.406–2.869). To test the potentially harmonic nature of this relationship, we multiplied each individual's alpha peak frequency with factor 2 to get the estimated harmonic beta peak (mean, 20.3 Hz; SD, 2.3 Hz), and compared this with the actual beta peak frequency (mean, 20.2 Hz; SD, 3.1 Hz) using a paired-sample t-test. There was no significant difference between the two beta-estimates (t(31) = 0.139, p = 0.89), suggesting that beta peak frequency as observed during rest in the parietal cortex could be a second harmonic of alpha. However, note that for the other conditions, we found no sign of correlation between alpha and beta peak frequencies (light blue data points in Fig. 6D), which argues against a simple harmonic relationship.

## Discussion

Here we studied variability in individual alpha peak frequency both within and across subjects, in the occipital and parietal cortex under different experimental conditions, in 51 human subjects using source-reconstructed MEG. The average alpha peak frequency across subjects, conditions, and ROIs was 10.3 Hz with a within-subject SD of 0.9 Hz and a between-subject SD of 2.8 Hz. In both regions we observed an increase of alpha peak frequency from resting state and passive visual stimulation conditions to the N-back paradigm (approximately 1 Hz increase), with a significantly higher alpha peak frequency in the 2-back compared to the 0-back condition. The increase in alpha peak frequency for the N-back task seemed to be strongest in the occipital cortex, but evidence for a significant ROI interaction was inconclusive. We observed a significant positive correlation between parietal alpha and beta peak frequencies during rest, suggestive of a harmonic relationship. However, the correlation disappeared in other conditions, and was not observed for the occipital ROI.

### Intra-subject variability: increase of alpha frequency with engagement

The increase in alpha frequency from rest to N-back conditions seems to be related to active engagement of the system. Since no increase in alpha frequency was observed for the passive visual stimulation condition, we can exclude that stimulation per se (and the accompanying evoked response) caused an apparent frequency shift. Moreover, the additional increase from the 0-back to the more demanding 2-back task further supports an active engagement or cognitive demand interpretation.

(Note that the latter effect was accompanied by a decrease in alpha power. This load-dependent decrease of alpha power has been reported before for the N-back paradigm (Gevins et al., 1997; Pesonen et al., 2007) and contrasts with the load-dependent alpha power increase that has been reported for the Sternberg paradigm and visuospatial WM tasks (Jensen et al., 2002; Roux et al., 2012; Sauseng et al., 2009; Tuladhar et al., 2007). It has been suggested that this dissociation between WM paradigms can be explained by the different nature of the tasks: in the latter tasks, increased alpha power during retention is interpreted to reflect suppression of distracting input to prevent interference with the WM trace, while in the N-back paradigm decreased alpha power is thought to reflect active engagement since encoding, retention and decision processes overlap in time here.)

What could the functional role be of the increase in frequency with increasing task demands? Given the functional inhibition hypothesis of alpha, the lower the frequency the longer the windows of phasic suppression (Haegens et al., 2011b; Jensen et al., 2012). This may be beneficial during rest, but active engagement in a task context may require different parameters. Or perhaps the frequency shift simply reflects different neuronal populations being activated, depending on task demands (Sadaghiani et al., 2010). These different networks may run on slightly different alpha frequencies (e.g., due to differences in physiological makeup), and their differential activation may result in the observed shift. Whether this means there are indeed different alpha rhythms (Başar, 2012; Klimesch, 1999), or rather that there is an operating range within which these networks function, remains to be seen. Future research using intracranial methods (e.g., local field potential recordings in non-human primates, electrocorticographic recordings in surgical epilepsy patients, laminar recordings) to measure more closely the population activity of local networks under changing task conditions would be imperative to answer this question: is it the same networks producing different rhythms, or do different networks run on slightly different alpha frequencies?

Similarly, the positive (potentially harmonic) correlation between alpha and beta peak frequencies observed in the parietal cortex during rest, which was not apparent in other conditions, may reflect different networks being activated. The lack of beta modulation across conditions while alpha frequency increased, suggests that they are largely independent. Previous studies that showed (quasi-)harmonic relationships also did so only under resting-state conditions (Carlqvist et al., 2005; Gaarder and Speck, 1967; Nikulin and Brismar, 2006; Van Albada and Robinson, 2013). Computational modeling may provide further insight, as Jones et al. (2009) showed for the somatosensory system how the same neural networks, dependent on timing of feedforward and feedback inputs, can generate both alpha and beta rhythms that are interdependent but not strictly harmonic (nor fully overlapping in time). Perhaps a similar mechanism is at play for posterior alpha and beta dynamics — such a model could potentially explain differences in alpha/beta patterns between conditions as well as between subjects.

### Inter-subject variability

As expected, inter-subject variability exceeded intra-subject variability. Individual alpha peak frequency values were found including the entire search range of 7–14 Hz, with a between-subject SD of 2.8 Hz (note that the fact that we find a higher SD than previously reported may be due to the use of various tasks and more subjects). Thus, using a fixed narrow alpha range (say 8–12 Hz) to analyze all subjects would bias against those with an alpha peak frequency outside of that range. Even limiting our search range to 7–14 Hz may have precluded alpha peak detection in some participants. A small subset of subjects did not present a systematic alpha peak but had a prominent beta peak instead. The question remains whether 1) these subjects truly do not have an alpha rhythm, or 2) that we simply did not pick up alpha activity in these cases due to e.g. lack in SNR or suboptimal orientation of their dipoles (i.e., anatomical variation) for the MEG measurement, or 3) that their “alpha” rhythm runs at a frequency outside our search range.

### Occipital vs. parietal alpha

The posterior alpha activity observed across conditions was driven by both occipital and parietal sources. Given the limited spatial resolution of MEG, we cannot be sure that our occipital and parietal signals were fully separated (i.e., there may have been residual mixing of signals even after source reconstruction), although we did observe a trend of divergence between occipital and parietal alpha peak frequency during the N-back tasks. Previous studies have claimed regional differences in alpha peak frequency, however, most of this work was based on sensor-level EEG studies, with even further limited spatial resolution (e.g., Klimesch et al., 1993).

In any case, given the distribution of maximum alpha power localization across the occipital and parietal cortices, it appears that posterior alpha as observed on scalp level has a widespread origin. It is likely that this reflects summation of several local network rhythms. Whether these networks interact and whether they receive (driving or modulating) inputs from the same sources (e.g., thalamic nuclei and frontal control regions) remain important questions for future research.

### Conclusion

The alpha rhythm operates across a wider frequency range than the 8–12 Hz band many studies tend to include in their analysis. Using a fixed, limited frequency band might bias against certain subjects and conditions. Furthermore, we showed that alpha peak frequency increases with cognitive demands and task engagement, which should be taken into account when comparing power values between different conditions (i.e., power differences could be confounded with frequency shifts). We propose future research looking into this flexibility of the alpha rhythm, using intracranial methods to determine whether frequency shifts originate from within a population or are due to engagement of different networks.

## Figures and Tables

**Table 1:** Individual alpha peak frequency. Showing per condition and ROI: mean ± standard deviation (N). N indicates number of subjects that had a detectable peak for that particular condition ∗ ROI combination.

	Occipital ROI	Parietal ROI
Rest	9.981 ± 1.088 (47)	10.143 ± 1.147 (49)
Base	9.916 ± 1.303 (50)	10.075 ± 1.107 (51)
Stim	10.102 ± 1.249 (51)	10.108 ± 1.092 (51)
0-back	10.716 ± 1.412 (49)	10.304 ± 1.344 (51)
2-back	11.118 ± 1.132 (50)	10.816 ± 1.311 (50)
